# A 90-Day Safety Study of Meat from *MSTN* and *FGF5* Double-Knockout Sheep in Wistar Rats

**DOI:** 10.3390/life12020204

**Published:** 2022-01-29

**Authors:** Yue Zhao, Mingming Chen, Yao Li, Xueling Xu, Sujun Wu, Zhimei Liu, Shiyu Qi, Guang Yi, Xiaosheng Zhang, Jinlong Zhang, Xiaofei Guo, Kun Yu, Shoulong Deng, Yan Li, Zhengxing Lian

**Affiliations:** 1 Beijing Key Laboratory for Animal Genetic Improvement, National Engineering Laboratory for Animal Breeding, Key Laboratory of Animal Genetics and Breeding of the Ministry of Agriculture, College of Animal Science and Technology, China Agricultural University, Beijing 100193, China; zhaoyuetx@cau.edu.cn (Y.Z.); chenmingming1937@cau.edu.cn (M.C.); BS20183040368@cau.edu.cn (Y.L.); BS20203040330@cau.edu.cn (X.X.); amywsj@cau.edu.cn (S.W.); S20193040539@cau.edu.cn (Z.L.); s20203040579@cau.edu.cn (S.Q.); sy20203040712@cau.edu.cn (G.Y.); yukun@cau.edu.cn (K.Y.); 2 Institute of Animal Husbandry and Veterinary Medicine, Tianjin Academy of Agricultural Sciences, Tianjin 300381, China; zhangxs0221@126.com (X.Z.); jlzhang1010@163.com (J.Z.); guoxfnongda@163.com (X.G.); 3 NHC Key Laboratory of Human Disease Comparative Medicine, Institute of Laboratory Animal Sciences, Chinese Academy of Medical Sciences and Comparative Medicine Center, Peking Union Medical College, Beijing 100021, China

**Keywords:** meat, *MSTN*, *FGF5*, sheep, 90-day feeding, rat

## Abstract

*MSTN* and *FGF5* gene knockout sheep generated by the CRISPR/Cas9 system exhibit the ‘double-muscle’ phenotype, and increased density and length of hairs, providing valuable new breeding material. In a previous study, we obtained *MSTN* and *FGF5* double-knockout sheep of significant breeding value. In this study, we carried out a 90-day feeding study in Wistar rats to assess the safety of genome-edited mutton. Seven rat groups with 10 females and 10 males per group were fed different concentrations (3.75%, 7.5%, and 15%) of double-knockout mutton or wild-type mutton in a conventional commercial diet for 90 days. At the end of the feeding, routine urine and blood tests and measurements of blood biochemical indicators were performed. Furthermore, the major organs of each group of rats were weighed and examined histopathologically. Although there were significant differences among the groups in some parameters, all values were within the normal ranges. Therefore, the 90-day rat feeding study showed that the meat from *MSTN* and *FGF5* double-knockout sheep did not have any long-term adverse effects on rat health. This study also provides valuable reference information for assessing the safety of meat from animals with knockout of multiple genes.

## 1. Introduction

Myostatin (*MSTN*), also called growth and differentiation factor-8 (*GDF-8*), is a member of the transforming growth factor-β (TGF-β) superfamily that negatively regulates skeletal muscle development and growth. Natural mutations in the *MSTN* gene can lead to muscle hypertrophy and thus a double-muscle phenotype. Belgian cattle are naturally occurring ‘double-muscled’ cattle, resulting from natural mutations in the *MSTN* gene, with well-developed muscle groups in the hips, thighs, chest, and forelimbs [1]. Various *MSTN*-KO animals (mouse, pig, sheep, and cattle) produced using genome editing techniques also exhibit a distinct double-muscle phenotype [2,3,4,5]. Fibroblast growth factor 5 (*FGF5*), a member of the fibroblast growth factor (*FGF*) superfamily, has the ability to inhibit the activation and proliferation of hair papillae and effectively inhibit hair growth and development [6]. Natural mutations in the human *FGF5* gene cause abnormal growth of eyelashes and body hair [7]. Furthermore, natural mutations in the *FGF5* locus in cats and dogs also result in increases in their coat length [8,9]. Upon the use of genome editing technology to produce *FGF5*-KO goats and sheep, both groups of animals exhibited increased hair length and density [10,11]. In summary, mutating the *MSTN* and *FGF5* genes can increase the economic value of large agricultural animals. *MSTN* knockout sheep and *FGF5* knockout sheep were produced in our lab previously and showed excellent meat and wool production, respectively [2,10]. Based on the role of *MSTN* with the *FGF5* gene, we generated CRISPR/Cas9-mediated *MSTN* and *FGF5* double-knockout sheep through microinjection. Both sgRNAs targeted the third exon of *MSTN* and *FGF5* genes, resulting in a cysteine deletion and a frameshift mutation in genome edited sheep, respectively, which displayed an obvious ‘double-muscle’ phenotype and increased wool length phenotype. The F1 generation of *MSTN* and *FGF5* knockout sheep were obtained by breeding the original *MSTN* and *FGF5* knockout rams with wild type ewes. All F1 generation sheep were identified as showing a similar phenotype to the original generation, with genotypes *MSTN*^+/−^ and *FGF5*^+/−^.

However, at the time of focusing on the value of genome edited animals, there are still ongoing controversies and concerns about the adverse effects of consuming genome editing-related foods on human health. When new genome editing-related products are developed, testing must be performed to determine whether the new trait will affect the nutritional value of the product or consumer health. Substantial equivalence embodies the concept that if a new food or food component is found to be substantially equivalent to an existing food or food component, it can be treated in the same manner with respect to safety. In recent years, the concept of substantial equivalence is the principle of safety assessment for genetically modified (GM) products that is accepted in most countries. In order to assess the adverse effects of GM-related foods, a 90-day oral toxicity study in rodents has been recommended by EFSA [12], and was later deemed mandatory in Europe. Ninety-day oral toxicity studies of GM foods are also needed in the world. Such biosafety evaluation experiments have now been reported for GM-related products such as meat and milk [13,14,15]. Existing EU regulations, as interpreted by the Court of Justice of the European Union case C-528/16iv, do not differentiate between TG and gene edited applications in agriculture. Therefore, the health assessment of rats that have consumed GM-related and gene editing-related products for 90 days is an important tool to assess the safety of food products.

To date, many animals with *MSTN* and *FGF5* mutations have been generated, but most of these studies evaluated their phenotype and explored related molecular mechanisms, while little attention has been paid to evaluating their biosafety. Thus far, no assessment of the risk of potential sub-chronic toxicity of *MSTN*-KO sheep meat or *FGF5*-KO sheep meat has been reported. Therefore, to promote the industrialization of genome-edited large livestock and to guarantee their safety for human consumption, the evaluation of the biosafety of products is essential.

To investigate the safety of meat from *MSTN* and *FGF5* double-knockout sheep, we conducted a 90-day feeding test on rats, in line with the National Standard for Food Safety 90-day oral toxicity test (Chinese Standard GB 15193.13-2015). The 90-day safety study in Wistar rats was carried out as a biosafety evaluation that is essential for animal products intended for direct human consumption and necessary to facilitate their commercialization. All rats were healthy and active, which provided evidence that meat derived from *MSTN* and *FGF5* double-knockout sheep is equivalent to meat derived from wild-type sheep.

## 2. Materials and Methods

All experimental animal protocols in this study were approved and performed in accordance with the requirements of the Animal Care and Use Committee at China Agricultural University (AW72011202-1-7). All surgeries were performed under sodium pentobarbital anesthesia, and all efforts were made to minimize any suffering experienced by the animals used in this study.

### 2.1. Sources of Sheep Meat

Four half-sibling F1 *MSTN* and *FGF5* double-knockout Dorper ewes and three wild-type Dorper ewes of similar ages and conditions were selected to collect meat samples from the front and hind legs of each sheep. All excess fat and connective tissue was removed from these meat samples. All samples of *MSTN* and *FGF5* double-knockout lamb were mixed together, as were all samples of wild-type conventional lamb. Both of these pooled samples were ground into a meat mixture using a meat grinder, further mixed thoroughly with other proportioned ingredients in a blender, and finally prepared as pellet feed that could be consumed by rats.

### 2.2. Diet Composition

The supplied diet met the nutritional standards for laboratory animals as outlined in GB 14924-2010 [16]. The meat from *MSTN* and *FGF5* double-knockout sheep and the meat from wild-type sheep were added to the formulated feed at 3.75%, 7.5%, and 15%, after which the main nutrient levels were matched and further processed into pelleted feed. The nutrient levels of the diets in all groups were sufficient for the growth and development of the rats (Table 1). After sterilization with ^60^Co radiation, the ration met the criteria for a clean rat feeding ration.

### 2.3. Animals and Feeding Doses

A total of 140 clean Wistar rats, weighing 80–100 g, were selected and purchased from Beijing Vital River Laboratory Animal Technology Co. (SCXK (Jing) 2016-0006). The 90-day study was carried out in the Animal House of the Ministry of Agriculture and Rural Affairs, Agricultural Products Quality Supervision, Inspection and Testing Centre (Beijing), under specific pathogen free (SPF) conditions (SYXK (Jing) 2020-0052). The rats were housed in an environmental controlled room with the temperature at 20–24 °C, 40–70% relative humidity, a 12 h artificial light/dark cycle, and 15 air changes/h. They were provided food and water *ad libitum* at all times during the adaptive phase and the formal testing period. After 5 days of the adaptive phase, the animals were randomly divided into seven groups of 20 rats each, with equal numbers of males and females, with matching between the groups for sex and body weight. Diets mixed with 3.75%, 7.5%, and 15% mutton were given to the rats for 90 days (Table 2). 

### 2.4. Indicator Testing

#### 2.4.1. Appearance, Weight, Food Intake, and Ocular Examinations

The rats were observed daily in terms of activity, coat color, feeding and excretion, and signs of poisoning and death. The rats were also observed weekly for growth and development; food intake and body weight were also recorded. At the end of the feeding, final body weight, body weight gain, and feed consumption were calculated. Before the test and at the end of the test, ocular (cornea, lens, bulbar conjunctiva, and iris) examinations were performed on the experimental animals in the T3 group, N3 group, and CK group.

#### 2.4.2. Routine Blood Tests and Blood Biochemistry

At the 90th day of feeding, the rats were fasted for 12 h, anesthetized, and underwent blood sampling from the inner canthus of the eye. Anticoagulated blood was collected for determination of white blood cell (WBC) count, red blood cell (RBC) count, hemoglobin (HGB), hematocrit (HCT), and platelet count (PLT) using an automatic hematology analyzer HEMAVET950 (Drew Scientific, Miami, FL, USA). Prothrombin time (PT) and activated partial thromboplastin time (APTT) were evaluated on an automatic coagulation analyzer CA-1500 (Sysmex, Kobe, Japan). Blood samples for biochemistry were collected into tubes containing no anticoagulant and centrifuged at 3000 rpm for 15 min to obtain serum. The serum was assayed using an automatic biochemistry analyzer HITACHI 7020 (Hitachi High-Technologies Corporation, Tokyo, Japan) to obtain biochemical parameters such as alanine aminotransferase (ALT), aspartate aminotransferase (AST), alkaline phosphatase (ALP), glutamyl transpeptidase (GGT), urea, creatinine (Cr), glucose (Glu), total protein (TP), albumin (Alb), total cholesterol (TC), triglycerides (TG), chloride (Cl), potassium (K), and sodium (Na).

#### 2.4.3. Routine Urine Tests

On the 90th day of feeding, the urine of the experimental animals was collected for routine examinations (e.g., urine protein, relative density, pH, glucose, and occult blood).

#### 2.4.4. Weighing of Organs

On the 90th day of feeding, the rats were dissected for gross pathology. Their brain, heart, thymus, adrenal glands, liver, kidney, spleen, testes, epididymis, uterus, and ovaries were isolated. The absolute weights of these organs were recorded and their relative weights (organ/body ratio) were calculated.

#### 2.4.5. Histopathology

Tissues from the brain, pituitary, thyroid, thymus, lung, heart, liver, spleen, kidney, adrenal gland, stomach, duodenum, jejunum, ileum, cecum, colon, rectum, pancreas, mesenteric lymph nodes, ovary, uterus, testis, epididymis, prostate, and bladder of the experimental rats were immersed in 4% neutral formalin fixative. These samples were made into paraffin tissue sections and were routinely stained with HE [17]. Histopathological analysis was performed under a microscope to observe the differences between the experimental and control groups.

### 2.5. Statistical Analysis

All values are presented as mean ± SD. SPSS 26.0 statistical software (IBM Corporation, Somers, NY, USA) was used for statistical analysis. After analysis of homogeneity of variance, one-way ANOVA was performed, and Tukey’s multiple comparison test was used to analyze the significance of pairwise differences. *p* < 0.05 was considered statistically significant.

## 3. Results

### 3.1. Clinical Signs

During the 90-day feeding period, all rats were active, with normal shiny coats; no abnormal nose, eye, or mouth discharges; and no abnormal excretions. No deaths due to toxicity were observed in any group. The ocular examinations (corneal, lens, bulbar conjunctiva, and iris) of the animals in the T3 group, N3 group, and CK group produced no abnormal results at the start and end of the test.

### 3.2. Body Weight and Food Consumption

**Figure 1:**
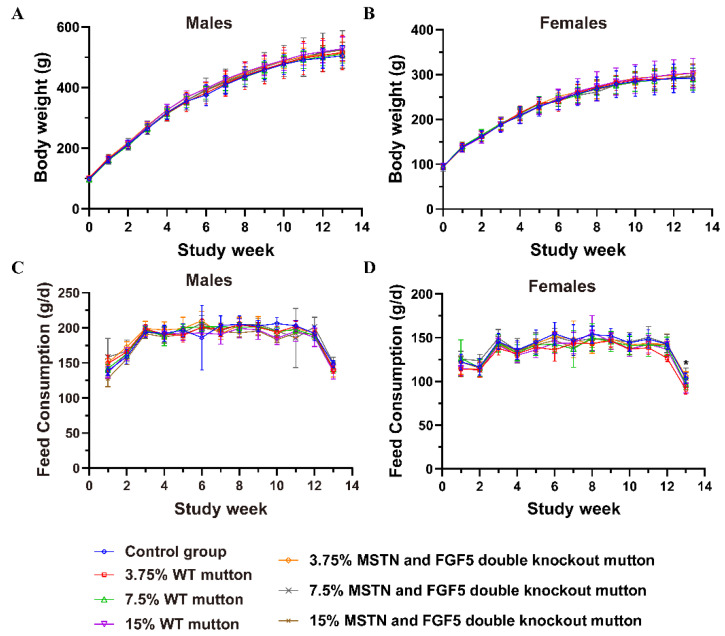
Body weight, feed consumption and feed conversion rate of male and female rats. (**A**) Mean weekly body weights of male rats. Line plots of mean male body weights (g) per group. (**B**) Mean weekly body weights of female rats. Line plots of mean female body weights (g) per group. (**C**) Mean weekly feed consumption of male rats. Line plots of mean feed consumption per group (g) and per week in the case of male rats. (**D**) Mean weekly feed consumption of female rats. Line plots of mean feed consumption per group (g) and per week in the case of female rats. * indicate significant differences (*p* < 0.05) between 3.75% WT mutton and 3.75% MSTN and FGF5 double knockout mutton.

The body weights of rats consuming different contents of the *MSTN* and *FGF5* double-knockout mutton during the 90-day feeding period were not significantly different from those of the corresponding wild-type control mutton and CK groups (*p* ≥ 0.05) (Figure 1A,B). Compared with the corresponding wild-type control mutton groups, there was no significant difference in the amount of meat eaten at most time points in the gene-edited mutton group, but the amount of food eaten by female rats in week 13 was significantly higher in the T1 group than in the N1 group (*p* < 0.05) (Figure 1C,D). Additionally, body weight gain and total food consumption of the rats in each test group were not significantly different from those of the corresponding wild-type control mutton groups and CK control groups (*p* ≥ 0.05) (Table 3).

### 3.3. Routine Blood Tests

Compared with those of the corresponding wild-type control mutton groups, the results of routine blood tests of the *MSTN* and *FGF5* double-knockout mutton groups were not significantly different (*p* ≥ 0.05) (Table 4). There were significant differences in individual indicators of routine blood tests between the *MSTN* and *FGF5* double-knockout mutton groups and the CK group (*p* < 0.05). However, these differences were not related to dose and were therefore not associated with feeding on *MSTN* and *FGF5* knockout meat.

### 3.4. Blood Biochemistry

In male rats, the AST, Alb, and LDH values in the T2 group and the TC values in the T3 group, were significantly different from those of the corresponding wild-type control mutton groups (*p* < 0.05). However, there was no significant difference compared to the CK group (*p* ≥ 0.05). In female rats, TP values in the T1 and T3 groups, AST, Alb, Cr, and TC values in the T3 group were significantly different from those of the corresponding wild-type control mutton groups (*p* < 0.05). However, there was no significant difference compared to the CK group (*p* ≥ 0.05). These differences in blood biochemistry were not dose-related and were within the normal range, suggesting that feeding on *MSTN* and *FGF5* knockout meat was not the cause of these differences (Table 5).

### 3.5. Routine Urine Tests

Among the results of routine urine tests, there were no significant differences between the *MSTN* and *FGF5* double-knockout mutton groups and the control group (*p* ≥ 0.05) (Table 6). Some male rats in the N1, N3, and T1 groups had occult blood, which but this was randomly distributed among the groups, so occult blood was not related to feeding on *MSTN* and *FGF5* knockout meat. Positive ketone bodies were observed in the male group but not in the female group. Additionally, high protein indicators of routine urine tests were randomly observed in both sexes.

### 3.6. Organ Weights and Histopathology

Compared with the corresponding wild-type control mutton groups, there were no significant differences in the thymus weight and thymus relative weight (organ/body ratio) at most time points in the gene-edited mutton group, but there were significant differences between the T1 group and the N1 group (*p <* 0.05). The thymus weight and thymus relative weight (organ/body ratio) of male rats in the T1 group were significantly different (*p <* 0.05) compared with N1 group, but no differences of this type were observed in the high-dose group (T2, T3). Kidney weights in the T2 group, kidney coefficients in male rats in the T2 group, and adrenal coefficients in female rats in the T1 group were significantly lower than those in the corresponding wild-type control mutton groups (*p* < 0.05), but not significantly different compared with the CK group (*p* ≥ 0.05) (Table 7 and Table 8).

**Figure 2:**
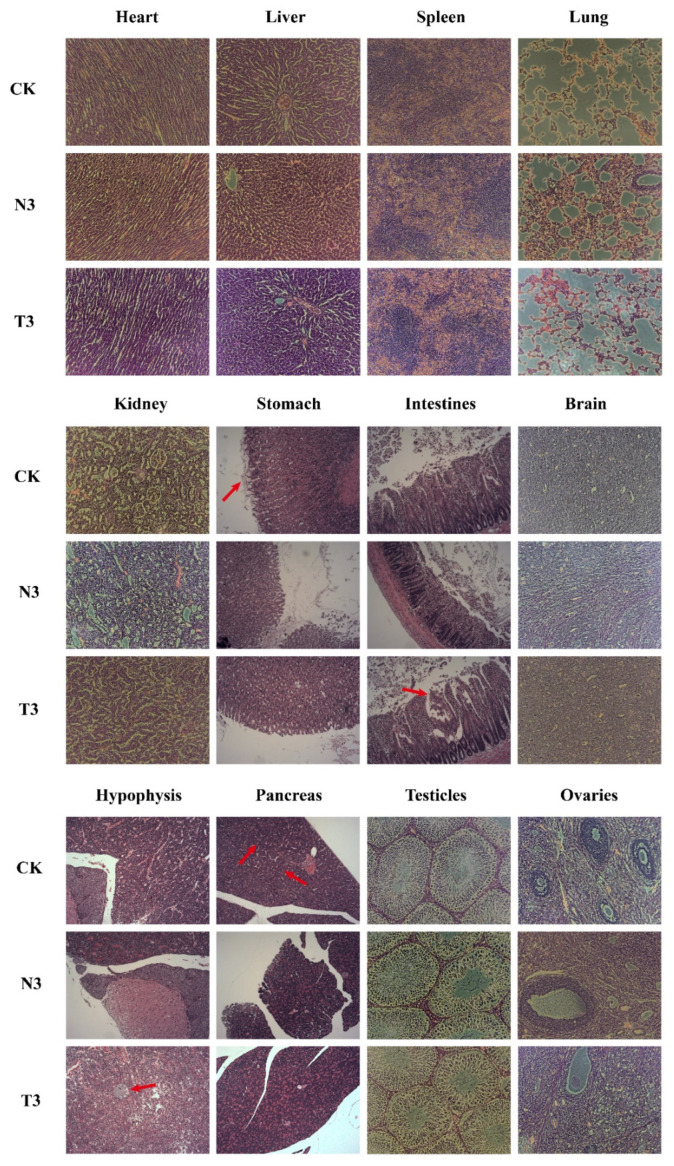
HE-stained sections of different tissues (100X). Group CK: fed a commercially available diet; Group N3: fed a formulated diet containing 15% wild-type control mutton; T3: fed a formulated diet containing 15% e *MSTN* and *FGF5* double knock-out mutton. Stomach: red arrow points to the location of the erosion. Intestine: red arrow points to the location of the erosion. Pancreas: red arrow points to the location of the intracellular lipid droplet. Pituitary: red arrow points to the location of the cyst.

Histopathological examination revealed that the male rats showed higher incidences of positive results than females in a number of tests, including lipid droplets in some cells of the pancreatic tissue, ileal mucosal erosion, and pituitary cysts, but no significant group differences were observed (Figure 2). Furthermore, mild lesions were observed in the stomach and duodenum in both female and male rats.

## 4. Discussion

Taking into account the balance of nutrients and energy in feed, the maximum proportion of mutton as a component of the feed was set at 15% in this study. The daily feed consumption of the rats was approximately 20 g, corresponding to a maximum daily intake of mutton of 3 g for each rat. Converting this value based on body surface area, an adult human weighting 60 kg would need to consume 192 g of mutton a day [18,19]. According to the “Dietary Guidelines for Chinese Residents” an adult needs to consume 50–75 g of meat per day [20]; clearly, 192 g is much higher than the normal human dietary intake. The minimum amount of mutton added to the rat feed in this study was set at 3.75%. This is equivalent to 64 g of mutton a day for a 60 kg adult human, which is in line with the dietary standards for Chinese residents. Previously, similar maximum additive levels were used in 90-day studies of rats feeding on lamb and pork [21,22].

During the 90-day feeding study, positivity for ketone bodies was observed in the male group, but not in the female group. There are two possible reasons for this: First, the use of fat to provide calories after starvation produces ketone bodies in the metabolic pathway, which are then excreted in the urine [23]. Second, in diabetic patients with high blood glucose, the body is unable to use blood glucose as a source of energy, but instead mobilizes body fat to provide energy, accelerating the breakdown of fat and producing excess ketone bodies, which are excreted in the urine [24,25]. In combination with the normal Glu values and blood glucose in blood biochemistry and routine urine tests, it is assumed that this may be related to the interruption of normal feeding during urine collection. Furthermore, proteinuria is a strong predictor of adverse kidney disease and important for the assessment and treatment of chronic kidney disease [26]. High protein indicators were present in the urine routine of only a few rats in each group, predicting kidney lesions and reduced renal filtration [27]. In addition, occult blood was observed in the urine of three groups of rats. Generally, there were no or few red blood cells in urine. The presence of blood in the urine is closely related with serious diseases, such as chronic nephrotic syndrome and urinary system tumors [28]. Further examination of the urinary system showed no stones or tumors, suggesting that inflammation may be present in the kidneys of some of the rats. In conjunction with pathological examination, some rats were diagnosed with chronic kidney disease. However, these rats with chronic nephritis were randomly distributed in each group. There were no significant differences between the groups, so positivity for ketone bodies, proteinuria and occult blood should not be related to the addition of genome-edited mutton to the diet. In the natural state, common pathological changes in the rat kidney may include pyelonephritis, calcium deposits in the renal tubules, and dilatation of the renal pelvis, with strain and sex being important factors in the incidence. We speculate that the rats in this experiment suffered from kidney disease due to inherent causes.

Upon analyzing liver function-related indicators, only AST and ALB were significantly different among the groups, but they were all within the normal range. Neither the relative weight nor the results of histological analysis concerning the liver differed between the various groups. Feeding on *MSTN* and *FGF5* double-knockout lamb also had no effect on liver function.

The presence of cysts in pituitary tissue could be common in experimental animals. The small lipid droplets within the cells of the pancreatic tissue may be associated with experimental manipulation. The stomach and duodenum also showed mild lesions, possibly related to the fixation procedure. These pathological changes were randomly distributed in each group. Feeding on *MSTN* and *FGF5* double-knockout lamb was not relevant to these changes.

In summary, in this 90-day subchronic study, meat derived from *MSTN* and *FGF5* double-knockout lamb exhibited no toxic effects on rats, appearing to be equivalent to meat derived from wild-type lamb. The results of this study are similar to those already reported for rats fed *MSTN* gene-edited pork for 90 days [22]. To further evaluate the safety of *MSTN* and *FGF5* double-knockout lamb, acute oral tests and protein heat stability tests may be required at a later stage. This experiment not only demonstrates the safety in terms of subtoxicity of *MSTN* and *FGF5* double-knockout lamb, but also sets a precedent for the safety of food related to multi-gene-edited animals, and could facilitate the industrialization of gene-edited agricultural animals with excellent potential.

## 5. Conclusions

In accordance with the National Standard for Food Safety 90-day oral toxicity test (Chinese Standard GB 15193.13-2015), a 90-day oral toxicity test using *MSTN* and *FGF5* double-knockout lamb was performed on rats. The results showed no obvious signs of poisoning or toxicity-related deaths in all the groups, and there were no statistically significant differences in body weight per week, body weight gain, total food consumption, and urine among the groups. Several statistically significant differences were observed in weekly food consumption, blood biochemistry, blood count, organ weight, and organ relative weights (organ/body ratio) compared with wild-type controls; however, these indicators were within the normal range. These differences should not be considered physiologically significant. No histopathological alterations associated with feeding on *MSTN* and *FGF5* double-knockout lamb were observed in the examined organs. Moreover, no adverse nutritional or toxic effects or unintended adverse effects of the *MSTN* and *FGF5* double-knockout mutton were observed in rats during the test period. The above analysis indicates that these differences were not associated with long-term consumption of feed containing the *MSTN* and *FGF5* double-knockout mutton. Therefore, we conclude that the *MSTN* and *FGF5* double-knockout mutton exhibited no toxic effects on rats when compared with its conventional comparators as presented in this 90-day subchronic study.

## Figures and Tables

**Table 1:** Main nutrient composition of feed for each group.

Ingredients	Wild-Type Mutton			*MSTN* and *FGF5* Double Knock-Out Mutton		
	3.75%	7.5%	15%	3.75%	7.5%	15%
Water content (g/100 g)	6.54	6.01	4.97	6.49	5.72	6.24
Ash content (g/100 g)	5.8	5.9	5.9	5.8	5.8	5.8
Crude protein (g/100 g)	20.5	18.4	19.1	20.3	19.5	19.1
Crude fat (g/100 g)	4.7	5.0	5.4	4.7	5.2	5.1
Crude fiber (g/100 g)	4.3	4.0	4.3	4.2	3.9	4.0
Ca (g/100 g)	1.14	1.11	1.11	1.13	1.10	1.04
*p* (g/100 g)	0.86	0.78	0.79	0.83	0.73	0.74

**Table 2:** Study design—dose groups.

Group	Diet	Number of Animals/Group	
		Male	Female
CK	commercially available diet	10	10
N1	3.75% wild-type control mutton	10	10
N2	7.5% wild-type control mutton	10	10
N3	15% wild-type control mutton	10	10
T1	3.75% *MSTN* and *FGF5* double-knockout mutton	10	10
T2	7.5% *MSTN* and *FGF5* double-knockout mutton	10	10
T3	15% *MSTN* and *FGF5* double-knockout mutton	10	10

**Table 3:** Summary of body weight gain and total food consumption.

Sex	Group	Body Weight Gain (g, *n* = 10)	Total Food Consumption (g, *n* = 10)	Total Food Utilization (%, *n* = 10)
Male rats	CK	406.9 ± 29.7	2426.8 ± 101.3	16.8 ± 1.0
	N1	413.8 ± 54.4	2425.1 ± 47.1	17.1 ± 2.1
	N2	413.3 ± 26.0	2417.7 ± 55.1	17.1 ± 0.8
	N3	429.0 ± 38.7	2362.2 ± 112.6	18.2 ± 1.4
	T1	410.8 ± 25.3	2450.9 ± 117.5	16.8 ± 1.0
	T2	426.5 ± 59.7	2446.9 ± 130.2	17.4 ± 2.0
	T3	425.1 ± 25.0	2346.4 ± 56.2	18.1 ± 1.0
Female rats	CK	196.9 ± 29.7	1811.9 ± 112.5	10.8 ± 1.4
	N1	197.3 ± 13.8	1698.3 ± 57.2	11.6 ± 0.8
	N2	195.4 ± 28.2	1755.2 ± 102.5	11.1 ± 1.2
	N3	207.6 ± 32.0	1736.0 ± 114.9	12.0 ± 1.6
	T1	210.8 ± 18.6	1770.3 ± 52.0	11.9 ± 1.2
	T2	202.0 ± 18.6	1814.0 ± 99.2	11.1 ± 1.1
	T3	204.6 ± 25.0	1764.1 ± 73.1	11.6 ± 1.4

**Table 4:** Summary of blood routines (*n* = 10, x ± SD).

Sex	Group	WBC (10^9^/L)	RBC (10^12^/L)	HCT (%)	HGB (g/L)	PLT (10^9^/L)	PT (s)	APTT (s)	NE (10^9^/L)	LY (10^9^/L)	EO (10^9^/L)	MO (10^9^/L)	BA (10^9^/L)
Male rats	CK	9.22 ± 2.31	7.17 ± 0.40	135 ± 6	44.0 ± 1.7	702 ± 80	9.2 ± 0.1	20.7 ± 1.4	3.41 ± 0.75	4.74 ± 1.37	0.02 ± 0.01	1.04 ± 0.25	0.00 ± 0.00
	N1	7.18 ± 1.37	7.17 ± 0.47	129 ± 7	42.0 ± 2.3	661 ± 84	9.3 ± 0.4	23.8 ± 5.2	2.84 ± 0.33	3.60 ± 1.20	0.05 ± 0.02	0.68 ± 0.20 *	0.00 ± 0.00
	N2	7.98 ± 3.22	7.44 ± 0.46	136 ± 8	45.0 ± 2.6	637 ± 55	9.4 ± 0.2	26.0 ± 3.6 *	3.10 ± 0.98	3.96 ± 1.96	0.06 ± 0.06	0.85 ± 0.39	0.01 ± 0.02
	N3	7.06 ± 2.18	7.14 ± 0.29	129 ± 8	42.2 ± 2.1	692 ± 50	9.6 ± 0.3 *	24.6 ± 2.4 *	2.61 ± 0.75	3.62 ± 1.46	0.03 ± 0.01	0.80 ± 0.25	0.00 ± 0.01
	T1	8.28 ± 2.11	7.06 ± 0.52	129 ± 10	41.9 ± 3.3	680 ± 80	9.7 ± 0.6	25.7 ± 3.6 *	3.23 ± 0.86	4.22 ± 1.30	0.04 ± 0.02	0.78 ± 0.26	0.00 ± 0.01
	T2	9.49 ± 2.04	7.56 ± 0.57	138 ± 11	44.9 ± 3.8	613 ± 75	9.7 ± 0.4 *	24.6 ± 2.4 *	3.41 ± 0.83	5.13 ± 1.23	0.06 ± 0.05	0.89 ± 0.17	0.01 ± 0.02
	T3	8.99 ± 1.78	7.53 ± 0.44	138 ± 9	44.5 ± 2.6	673 ± 72	9.7 ± 0.4 *	26.5 ± 2.5 *	3.55 ± 0.85	4.70 ± 1.01	0.06 ± 0.05	0.67 ± 0.20 *	0.01 ± 0.03
Female rats	CK	5.73 ± 1.89	6.81 ± 0.45	129 ± 11	43.4 ± 3.6	637 ± 65	9.6 ± 0.5	24.6 ± 5.1	1.56 ± 0.29	3.77 ± 1.72	0.01 ± 0.01	0.39 ± 0.11	0.00 ± 0.00
	N1	5.65 ± 1.53	6.63 ± 0.74	129 ± 17	42.3 ± 5.4	686 ± 95	9.4 ± 0.6	22.5 ± 4.3	1.77 ± 0.44	3.43 ± 1.03	0.03 ± 0.02	0.42 ± 0.15	0.00 ± 0.00
	N2	6.43 ± 1.50	6.43 ± 0.31	123 ± 9	41.7 ± 2.9	712 ± 86	9.6 ± 0.9	23.8 ± 3.6	1.90 ± 0.35	4.07 ± 1.18	0.02 ± 0.02	0.43 ± 0.15	0.00 ± 0.00
	N3	5.87 ± 1.85	6.62 ± 0.47	131 ± 9	43.1 ± 3.2	652 ± 76	9.7 ± 0.5	25.2 ± 5.1	1.90 ± 0.53	3.50 ± 1.30	0.02 ± 0.02	0.45 ± 0.13	0.01 ± 0.02
	T1	5.35 ± 1.37	6.32 ± 0.49	120 ± 11	40.6 ± 3.7	699 ± 70	9.6 ± 0.5	25.2 ± 4.3	1.47 ± 0.42	3.49 ± 1.13	0.02 ± 0.02	0.37 ± 0.11	0.01 ± 0.02
	T2	5.83 ± 1.30	6.56 ± 0.42	124 ± 6	42.0 ± 2.3	654 ± 42	9.7 ± 0.7	24.1 ± 2.7	1.78 ± 0.37	3.62 ± 1.02	0.02 ± 0.01	0.41 ± 0.06	0.00 ± 0.00
	T3	5.43 ± 0.87	6.16 ± 0.49	123 ± 9	39.7 ± 3.0	689 ± 74	9.9 ± 0.5	24.4 ± 2.6	1.72 ± 0.34	3.28 ± 0.72	0.02 ± 0.01	0.42 ± 0.10	0.00 ± 0.00

Group CK: fed a commercially available diet; Group N1: fed a formulated diet containing 3.75% wild-type control mutton; Group N2: fed a formulated diet containing 7.5% wild-type control mutton; Group N3: fed a formulated diet containing 15% wild-type control mutton; Group T1: fed a formulated diet containing 3.75% *MSTN* and *FGF5* double knock-out mutton. Group T2: fed a formulated diet containing 7.5% *MSTN* and *FGF5* double knock-out mutton. Group T3: fed a formulated diet containing 15% *MSTN* and *FGF5* double knock-out mutton. WBC: white blood cell count; RBC: erythrocyte count; HCT: hematocrit; HGB: hemoglobin; PLT: platelet (thrombocyte) count; PT: prothrombin time; APPT: activated partial thromboplastin time; NE: neutrophil count; LY: lymphocyte count; EO: eosinophil count; MO: monocyte count. * *p* < 0.05 compared with the CK group.

**Table 5:** Summary of blood biochemistry (*n* = 10, x ± SD).

Gender	Group	ALT	AST	TP	Alb	ALP	Glu	Urea	Cr	TC	TG	GGT	LDH	K	Na	Cl
		(U/L)	(U/L)	(g/L)	(g/L)	(U/L)	(mmol/L)	(mmol/L)	(μmol/L)	(mmol/L)	(mmol/L)	(U/L)	(U/L)	(mmol/L)	(mmol/L)	(mmol/L)
Male rats	CK	58.9 ± 9.0	118 ± 16	61.2 ± 5.0	23.4 ± 2.0	138 ± 35	11.83 ± 1.76	9.37 ± 1.95	50.3 ± 8.7	3.07 ± 0.75	0.75 ± 0.24	1.89 ± 0.96	1349 ± 203	5.03 ± 0.39	135.7 ± 4.8	97.8 ± 4.5
	N1	59.4 ± 11.8	111 ± 16	63.9 ± 3.6	23.4 ± 1.9	128 ± 31	12.96 ± 3.41	7.79 ± 1.36	51.9 ± 9.3	2.96 ± 0.45	1.02 ± 0.47	1.43 ± 1.52	1044 ± 364	5.37 ± 1.13	136.1 ± 10.0	98.2 ± 9.8
	N2	61.9 ± 13.2	141 ± 22	64.8 ± 7.9	24.7 ± 2.4	129 ± 34	13.53 ± 2.99	8.90 ± 2.02	53.3 ± 12.0	2.88 ± 0.60	0.80 ± 0.33	1.07 ± 1.86	1363 ± 197	4.73 ± 0.32	135.9 ± 8.2	96.6 ± 7.5
	N3	45.0 ± 7.9	111 ± 15	54.4 ± 7.6	19.5 ± 1.8 *	106 ± 11	10.98 ± 2.18	7.66 ± 1.62	41.1 ± 4.7	2.18 ± 0.24	0.77 ± 0.42	2.70 ± 1.49	1274 ± 326	5.16 ± 0.38	139.6 ± 1.7	101.3 ± 2.7
	T1	44.7 ± 7.0	94 ± 13	56.3 ± 3.6	19.1 ± 2.1 *#	112 ± 26	11.01 ± 1.22	7.14 ± 0.89	40.9 ± 2.6	2.51 ± 0.50	0.54 ± 0.16	2.81 ± 0.69	945 ± 250 *	4.85 ± 0.34	140.3 ± 1.6	102.6 ± 2.5
	T2	49.8 ± 12.2	91 ± 14 #	57.8 ± 6.5	21.5 ± 1.6 #	122 ± 24	8.83 ± 1.21 *#	7.78 ± 1.22	41.1 ± 5.7	2.62 ± 0.26	0.65 ± 0.19	1.48 ± 1.67	981 ± 314 #	4.97 ± 0.30	141.3 ± 1.7	103.9 ± 3.5
	T3	57.9 ± 24.3	116 ± 46	59.3 ± 2.7	18.5 ± 2.8 *	106 ± 21	9.76 ± 1.75	7.40 ± 0.75	41.6 ± 5.9	2.66 ± 0.34 #	0.70 ± 0.22	1.09 ± 1.23	951 ± 249 *	5.31 ± 0.34	143.2 ± 11.3	105.7 ± 5.2 *
Female rats	CK	43.0 ± 6.5	89 ± 19	65.9 ± 10.5	26.6 ± 4.1	71 ± 17	8.42 ± 1.71	9.00 ± 0.90	47.6 ± 11.6	2.77 ± 0.79	0.57 ± 0.25	3.04 ± 0.93	968 ± 333	4.49 ± 0.29	139.9 ± 1.5	107.7 ± 3.2
	N1	44.1 ± 8.4	92 ± 18	57.7 ± 13.7	24.8 ± 4.0	79 ± 22	9.01 ± 2.91	8.79 ± 2.58	46.0 ± 11.6	2.70 ± 0.47	0.38 ± 0.08	1.70 ± 1.23	995 ± 404	4.47 ± 0.18	141.0 ± 2.0	107.5 ± 4.7
	N2	46.1 ± 6.8	93 ± 19	57.5 ± 6.8	23.7 ± 2.0	75 ± 20	9.32 ± 2.30	7.97 ± 0.85	40.8 ± 4.0	2.50 ± 0.57	0.50 ± 0.14	1.23 ± 1.09 *	994 ± 324	4.58 ± 0.34	141.6 ± 1.4	108.6 ± 3.4
	N3	57.3 ± 12.1	119 ± 29	73.9 ± 15.1	31.6 ± 4.8 *	89 ± 19	11.09 ± 1.71	9.20 ± 1.73	58.4 ± 10.7	3.69 ± 1.02 *	0.73 ± 0.26	2.67 ± 1.21	1473 ± 540	4.43 ± 0.28	140.6 ± 2.1	108.1 ± 4.3
	T1	58.9 ± 12.9 *	121 ± 39	76.1 ± 10.5 #	29.0 ± 3.8	98 ± 30	12.00 ± 2.70 *	9.27 ± 2.41	54.3 ± 9.3	2.84 ± 0.62	0.45 ± 0.10	1.61 ± 0.64	1209 ± 412	4.97 ± 0.61	140.4 ± 1.3	109.3 ± 2.1
	T2	51.1 ± 15.4	89 ± 18	58.5 ± 7.9	22.9 ± 3.0	67 ± 12	10.92 ± 2.76	8.15 ± 0.91	41.8 ± 6.5	2.35 ± 0.42	0.36 ± 0.07	2.08 ± 0.98	708 ± 122	4.59 ± 0.27	146.5 ± 12.4	113.9 ± 12.7
	T3	44.0 ± 12.4	80 ± 10 #	57.0 ± 8.2 #	21.9 ± 2.9 #	76 ± 32	8.90 ± 1.12	7.47 ± 1.18	43.0 ± 6.9 #	2.30 ± 0.59 #	0.52 ± 0.13	2.66 ± 1.28	919 ± 152	4.23 ± 0.33	140.5 ± 4.3	107.2 ± 5.4

Group CK: fed a commercially available diet; Group N1: fed a formulated diet containing 3.75% wild-type control mutton; Group N2: fed a formulated diet containing 7.5% wild-type control mutton; Group N3: fed a formulated diet containing 15% wild-type control mutton; Group T1: fed a formulated diet containing 3.75% *MSTN* and *FGF5* double knock-out mutton. Group T2: fed a formulated diet containing 7.5% *MSTN* and *FGF5* double knock-out mutton. Group T3: fed a formulated diet containing 15% *MSTN* and *FGF5* double knock-out mutton. ALT: alanine aminotransferase; AST: aspartate aminotransferase; TP: total protein; ALB: albumin; ALP: alkaline phosphatase; GLU: glucose; Cr: creatinine; TC: total cholesterol; TG: triglycerides; GGT: glutamyl transpeptidase; LDH: lactate dehydrogenase; K: potassium; Na: sodium; Cl: chlorine. # *p* < 0.05 compared with each conventional meat group. * *p* < 0.05 compared with the CK group.

**Table 6:** Summary of routine urine tests (*n* = 10, x ± SD).

Sex	Group	Ketone Body (mmol/L)	Protein (g/L)	Glucose (mmol/L)	Urine Specific Gravity	Occult Blood (cell/µL)	pH
Male rats	CK	0.2 ± 0.2	0.22 ± 0.30	0 ± 0	1.017 ± 0.003	0.0 ± 0.0	7.8 ± 0.3
	N1	0.1 ± 0.2	0.03 ± 0.06	0 ± 0	1.013 ± 0.003	2.5 ± 7.9	7.7 ± 0.3
	N2	0.2 ± 0.2	0.39 ± 0.43	0 ± 0	1.014 ± 0.003	0.0 ± 0.0	7.8 ± 0.4
	N3	0.0 ± 0.0	0.02 ± 0.05	0 ± 0	1.013 ± 0.003	1.0 ± 3.2	7.8 ± 0.3
	T1	0.2 ± 0.3	0.09 ± 0.13	0 ± 0	1.015 ± 0.003	9.0 ± 25.1	7.8 ± 0.3
	T2	0.2 ± 0.3	0.11 ± 0.14	0 ± 0	1.013 ± 0.003	0.0 ± 0.0	7.5 ± 0.3
	T3	0.1 ± 0.2	0.16 ± 0.32	0 ± 0	1.015 ± 0.003	0.0 ± 0.0	7.7 ± 0.3
Female rats	CK	0.0 ± 0.0	0.36 ± 0.94	0 ± 0	1.018 ± 0.005	0.0 ± 0.0	7.5 ± 0.9
	N1	0.0 ± 0.0	0.15 ± 0.32	0 ± 0	1.014 ± 0.004	0.0 ± 0.0	7.5 ± 0.4
	N2	0.0 ± 0.0	0.02 ± 0.05	0 ± 0	1.014 ± 0.003	0.0 ± 0.0	7.4 ± 0.3
	N3	0.0 ± 0.0	0.20 ± 0.42	0 ± 0	1.015 ± 0.004	0.0 ± 0.0	7.3 ± 0.4
	T1	0.0 ± 0.0	0.08 ± 0.13	0 ± 0	1.014 ± 0.003	0.0 ± 0.0	7.3 ± 0.4
	T2	0.0 ± 0.0	0.03 ± 0.09	0 ± 0	1.013 ± 0.003	0.0 ± 0.0	7.4 ± 0.3
	T3	0.0 ± 0.0	0.12 ± 0.31	0 ± 0	1.014 ± 0.004	0.0 ± 0.0	7.2 ± 0.3

Group CK: fed a commercially available diet; Group N1: fed a formulated diet containing 3.75% wild-type control mutton; Group N2: fed a formulated diet containing 7.5% wild-type control mutton; Group N3: fed a formulated diet containing 15% wild-type control mutton; Group T1: fed a formulated diet containing 3.75% *MSTN* and *FGF5* double knock-out mutton. Group T2: fed a formulated diet containing 7.5% *MSTN* and *FGF5* double knock-out mutton. Group T3: fed a formulated diet containing 15% *MSTN* and *FGF5* double knock-out mutton.

**Table 7:** Summary of organ weight (*n* = 10, x ± SD).

Sex	Group (g)	Brain (g)	Liver (g)	Spleen (g)	Heart (g)	Thymus (g)	Kidney (g)	Adrenal (g)	Testicles (Male) Ovaries (Female) (g)	Epididymis (Male) Uterus (Female) (g)
Male rats	CK	2.09 ± 0.10	13.51 ± 1.69	0.95 ± 0.16	1.33 ± 0.10	0.79 ± 0.27	3.22 ± 0.35	0.065 ± 0.010	3.90 ± 0.52	1.49 ± 0.24
	N1	2.09 ± 0.08	15.31 ± 2.59	0.88 ± 0.12	1.49 ± 0.19	0.82 ± 0.20	3.97 ± 0.30 *	0.068 ± 0.006	3.60 ± 0.50	1.41 ± 0.13
	N2	2.11 ± 0.08	13.56 ± 1.68	1.00 ± 0.23	1.28 ± 0.13	0.62 ± 0.24	3.81 ± 0.38 *	0.069 ± 0.013	3.71 ± 0.32	1.44 ± 0.13
	N3	2.13 ± 0.06	14.59 ± 1.49	0.90 ± 0.11	1.41 ± 0.14	0.76 ± 0.12	3.67 ± 0.35	0.064 ± 0.019	3.78 ± 0.40	1.31 ± 0.13
	T1	2.22 ± 0.24	14.14 ± 1.23	0.98 ± 0.12	1.52 ± 0.11	0.52 ± 0.10 *#	3.69 ± 0.35 *	0.070 ± 0.009	3.91 ± 0.22	1.42 ± 0.11
	T2	2.12 ± 0.14	14.45 ± 1.84	1.00 ± 0.19	1.43 ± 0.19	0.60 ± 0.15	3.28 ± 0.30 #	0.067 ± 0.014	3.59 ± 0.34	1.31 ± 0.19
	T3	2.15 ± 0.08	14.32 ± 1.02	0.92 ± 0.12	1.34 ± 0.08	0.92 ± 0.17	3.33 ± 0.36	0.061 ± 0.014	3.93 ± 0.50	1.38 ± 0.12
Female rats	CK	1.88 ± 0.09	7.67 ± 1.15	0.60 ± 0.08	0.88 ± 0.12	0.33 ± 0.09	1.91 ± 0.23	0.085 ± 0.012	0.171 ± 0.033	0.52 ± 0.10
	N1	1.93 ± 0.11	8.35 ± 0.91	0.61 ± 0.11	0.91 ± 0.09	0.36 ± 0.06	2.05 ± 0.17	0.097 ± 0.009	0.149 ± 0.030	0.52 ± 0.20
	N2	1.92 ± 0.09	8.01 ± 0.50	0.60 ± 0.09	0.99 ± 0.08	0.41 ± 0.11	2.02 ± 0.13	0.082 ± 0.016	0.173 ± 0.048	0.54 ± 0.26
	N3	1.94 ± 0.07	7.67 ± 1.13	0.58 ± 0.05	0.97 ± 0.15	0.33 ± 0.10	1.88 ± 0.19	0.079 ± 0.014	0.143 ± 0.038	0.52 ± 0.10
	T1	1.93 ± 0.10	8.03 ± 0.68	0.61 ± 0.06	0.92 ± 0.06	0.38 ± 0.07	1.96 ± 0.12	0.081 ± 0.013	0.159 ± 0.038	0.51 ± 0.11
	T2	1.93 ± 0.11	8.27 ± 1.03	0.62 ± 0.09	0.91 ± 0.07	0.37 ± 0.10	1.97 ± 0.09	0.086 ± 0.015	0.169 ± 0.023	0.57 ± 0.15
	T3	1.93 ± 0.08	7.64 ± 0.73	0.62 ± 0.06	0.97 ± 0.14	0.38 ± 0.07	2.02 ± 0.15	0.085 ± 0.008	0.163 ± 0.041	0.54 ± 0.14

Group CK: fed a commercially available diet; Group N1: fed a formulated diet containing 3.75% wild-type control mutton; Group N2: fed a formulated diet containing 7.5% wild-type control mutton; Group N3: fed a formulated diet containing 15% wild-type control mutton; Group T1: fed a formulated diet containing 3.75% *MSTN* and *FGF5* double knock-out mutton. Group T2: fed a formulated diet containing 7.5% *MSTN* and *FGF5* double knock-out mutton. Group T3: fed a formulated diet containing 15% *MSTN* and *FGF5* double knock-out mutton. # *p* < 0.05 compared with each conventional meat group. * *p* < 0.05 compared with CK group.

**Table 8:** Summary of relative organ weight/body weight (*n* = 10, x ± SD).

Gender	Group	Brain (%)	Liver (%)	Spleen (%)	Heart (%)	Thymus (%)	Kidney (%)	Adrenal (%)	Testicles (Male) Ovaries (Female) (%)	Epididymis (Male) Uterus (Female) (%)
Male rats	CK	0.41 ± 0.02	2.67 ± 0.29	0.19 ± 0.03	0.26 ± 0.02	0.15 ± 0.05	0.64 ± 0.06	0.013 ± 0.002	0.77 ± 0.09	0.30 ± 0.06
	N1	0.41 ± 0.04	2.97 ± 0.40	0.17 ± 0.01	0.29 ± 0.02	0.16 ± 0.03	0.78 ± 0.11 *	0.013 ± 0.002	0.70 ± 0.10	0.28 ± 0.03
	N2	0.42 ± 0.02	2.65 ± 0.20	0.20 ± 0.04	0.25 ± 0.01	0.11 ± 0.06	0.75 ± 0.07 *	0.014 ± 0.002	0.73 ± 0.08	0.28 ± 0.03
	N3	0.41 ± 0.03	2.76 ± 0.22	0.17 ± 0.02	0.27 ± 0.02	0.14 ± 0.02	0.70 ± 0.05	0.012 ± 0.003	0.72 ± 0.08	0.25 ± 0.03
	T1	0.43 ± 0.05	2.76 ± 0.29	0.19 ± 0.02	0.30 ± 0.02 *	0.10 ± 0.02 *#	0.72 ± 0.06	0.014 ± 0.002	0.77 ± 0.07	0.28 ± 0.02
	T2	0.41 ± 0.04	2.75 ± 0.19	0.19 ± 0.03	0.27 ± 0.03	0.11 ± 0.03	0.63 ± 0.04 #	0.013 ± 0.002	0.69 ± 0.09	0.25 ± 0.04
	T3	0.41 ± 0.02	2.74 ± 0.28	0.18 ± 0.02	0.26 ± 0.02	0.18 ± 0.03	0.64 ± 0.07	0.012 ± 0.003	0.75 ± 0.09	0.26 ± 0.02
Female rats	CK	0.65 ± 0.07	2.64 ± 0.35	0.21 ± 0.05	0.30 ± 0.02	0.11 ± 0.03	0.66 ± 0.05	0.029 ± 0.005	0.059 ± 0.013	0.18 ± 0.04
	N1	0.67 ± 0.04	2.87 ± 0.30	0.21 ± 0.03	0.31 ± 0.03	0.12 ± 0.02	0.71 ± 0.07	0.033 ± 0.003	0.051 ± 0.012	0.16 ± 0.10
	N2	0.66 ± 0.05	2.77 ± 0.18	0.21 ± 0.02	0.34 ± 0.02 *	0.14 ± 0.04	0.70 ± 0.06	0.028 ± 0.006	0.060 ± 0.019	0.19 ± 0.09
	N3	0.65 ± 0.07	2.53 ± 0.25	0.19 ± 0.02	0.32 ± 0.04	0.11 ± 0.03	0.62 ± 0.03	0.026 ± 0.005	0.048 ± 0.016	0.18 ± 0.05
	T1	0.64 ± 0.04	2.65 ± 0.13	0.20 ± 0.02	0.30 ± 0.01	0.13 ± 0.02	0.65 ± 0.05	0.027 ± 0.004 #	0.052 ± 0.010	0.17 ± 0.03
	T2	0.65 ± 0.04	2.80 ± 0.33	0.21 ± 0.03	0.31 ± 0.01	0.12 ± 0.03	0.67 ± 0.05	0.029 ± 0.006	0.057 ± 0.008	0.19 ± 0.04
	T3	0.65 ± 0.04	2.57 ± 0.17	0.21 ± 0.02	0.32 ± 0.03	0.13 ± 0.02	0.68 ± 0.04	0.029 ± 0.003	0.055 ± 0.017	0.18 ± 0.05

Group CK: fed a commercially available diet; Group N1: fed a formulated diet containing 3.75% wild-type control mutton; Group N2: fed a formulated diet containing 7.5% wild-type control mutton; Group N3: fed a formulated diet containing 15% wild-type control mutton; Group T1: fed a formulated diet containing 3.75% *MSTN* and *FGF5* double knock-out mutton. Group T2: fed a formulated diet containing 7.5% *MSTN* and *FGF5* double knock-out mutton. Group T3: fed a formulated diet containing 15% *MSTN* and *FGF5* double knock-out mutton. # *p* < 0.05 compared with each conventional meat group. * *p* < 0.05 compared with the CK group.
